# Management of recurrent stress urinary incontinence and urinary retention following midurethral sling insertion in women

**DOI:** 10.1308/003588412X13373405385610

**Published:** 2012-10

**Authors:** H Hashim, TR Terry

**Affiliations:** ^1^North Bristol NHS Trust,UK; ^2^University Hospitals of Leicester NHS Trust,UK

**Keywords:** Stress urinary incontinence, Recurrence, Treatment, Urinary retention

## Abstract

**INTRODUCTION:**

Synthetic midurethral slings are the most common operations performed for women with stress urinary incontinence (SUI). However, there is only very scarce evidence regarding the management of complications from these operations. The aim of this survey was to canvass expert opinion regarding the management of recurrent SUI and urinary retention following insertion of these slings.

**METHODS:**

Expert urologists and urogynaecologists in the UK with an interest in SUI were identified. Three clinical scenarios on recurrent SUI and one on urinary retention following midurethral sling placements were emailed twice to the experts.

**RESULTS:**

The majority of the experts chose a repeat synthetic midurethral retropubic transvaginal tape (TVT) as the procedure of choice for recurrent SUI in patients who had had a previous TVT or midurethral transobturator tape inserted. In patients who continued to suffer SUI after a failed second TVT, there were mixed results with experts choosing fascial slings, colposuspension and bulking agents as their preferred method of treatment. In women who develop urinary retention following a TVT, tape pull-down within two weeks was the preferred method among the experts. However, division of the tape within two to six weeks following the procedure was also popular.

**CONCLUSIONS:**

Based on expert opinion, it is difficult to make a recommendation as to the best method of treating recurrent SUI or urinary retention following tape insertion. There is an urgent requirement for well conducted, multicentre, randomised clinical trials to look at the management of these complications and also the tools used to assess the patient before salvage surgical management.

Stress urinary incontinence (SUI) is defined by the International Continence Society (ICS) as the complaint of involuntary loss of urine on effort or physical exertion including sporting activities, sneezing or coughing.^1^ The initial treatment of SUI in women includes a combination of pelvic floor muscle training and conservative measures such as weight loss and stopping smoking. These normally take about three months to work but in those who persevere with treatment, about 40% will see some improvement in symptoms. Some clinicians then might offer duloxetine, a norepinephrine serotonin reuptake inhibitor, which has been licensed in moderate to severe incontinence as an interim measure with some benefit.^2^

If duloxetine fails, the only active option available to treat the patient is surgery. Colposuspension has been the gold standard treatment for women with SUI for many years with long-term data of success. However, over the past several years it has been superseded gradually by synthetic midurethral slings, which now have 11-year data published on their success in treating SUI,^3^ and sling procedures have become the most common operation for SUI in women in England.^4^

There is evidence (level 1/2) that the retropubic transvaginal tape (TVT) is more effective than the Burch colposuspension and is equally as effective as traditional fascial sling operations. Operation time, hospital stay and time to resuming normal daily activity is shorter with the TVT than with colposuspension.^5^ Midurethral slings are about 80–90% successful in terms of cure rates. The main adverse effects are bladder perforation during surgery (5%), tape erosion (5%) and exacerbation of other lower urinary tract symptoms. Rarely, leg pain may be a feature of the inside-to-outside midurethral transobturator tape (TOT). The use of slings is not a contraindication in patients with overactive bladder syndrome/detrusor overactivity.

The prevalence of voiding dysfunction, including urinary retention, following midurethral slings ranges from 2–25% (level 2/3) with a surgical intervention required to resolve the problem in 0–5% of patients (level 2/3).^5^ However, there are no universally agreed criteria in making the diagnosis of urinary retention, which is based mainly on several clinical parameters. Diagnostic cystoscopy and multichannel invasive urodynamics have been used as adjuncts by some as an aid to make a diagnosis of outflow obstruction.

Currently, there are no randomised trials assessing the management of patients who suffer with recurrent SUI or urinary retention after midurethral sling surgery. The aim of this survey was therefore to canvass leading expert opinion regarding the management of these two common complications.

## Methods

Twenty-one urologists and twenty-one urogynaecologists were identified in the UK who were regarded as leading experts in the field of SUI. They were chosen from several databases including those of the ICS, British Society of Urogynaecology, British Association of Urological Surgeons and United Kingdom Continence Society. The choice was based on national and international reputation with regard to clinical activity, state-of-the-art lectures given, publications, research, and involvement in setting national and international guidelines.

Four clinical scenarios were sent via email to all the experts that covered the two complications of recurrent SUI and urinary retention following midurethral sling insertion (Table 1). A deadline for reply was given. Once the deadline had passed, the experts were sent a reminder email with a new deadline to increase the response rate. They were asked to answer each question with only one reply. The data were tabulated and analysed. Some experts replied with more than one answer for each scenario as this mimicked clinical real life practice (Table 2). The decision of one choice over the other depended on discussion with the patient and patient preference after careful counselling. All responses were taken into account in this case.

**Table 1:** Clinical scenarios

**Scenario 1**
**A lady has bothersome recurrent urodynamically proven stress incontinence following retropubic midurethral transvaginal tape insertion and has failed conservative and medical treatment. She has no detrusor overactivity and has normal detrusor voiding pressure. She would like treatment. Which is your next surgical approach?** *(Please choose one only.)*
Another synthetic retropubic transvaginal tape Synthetic transobturator tape Adjustable synthetic tape Rectus sheath sling Colposuspension Periurethral injection (eg Macroplastique®/collagen) Bladder neck closure and urinary diversion Other: _____________________________________________________________
**Scenario 2**
**A lady has bothersome recurrent urodynamic stress incontinence following transobturator midurethral tape insertion and has failed conservative and medical treatment. She has normal detrusor voiding pressure and no detrusor overactivity. She would like treatment. Which is your next surgical approach?** *(Please choose one only.)*
Retropubic synthetic transvaginal tape Synthetic transobturator tape Adjustable synthetic tape Rectus sheath sling Colposuspension Periurethral injection (eg Macroplastique®/collagen) Bladder neck closure and urinary diversion Other: _____________________________________________________________
**Scenario 3**
**A lady has bothersome recurrent urodynamic stress incontinence following *two* failed retropubic transvaginal tape insertions and has failed conservative and medical treatment. She has normal detrusor voiding pressure and no detrusor overactivity. She would like treatment. Which is your next surgical approach?** *(Please choose one only.)*
A third retropubic synthetic transvaginal tape Synthetic transobturator tape Adjustable synthetic tape Rectus sheath sling Colposuspension Periurethral injection (eg Macroplastique®/collagen) Bladder neck closure and urinary diversion Other: _____________________________________________________________
**Scenario 4**
**A lady has had midurethral synthetic mesh tape insertion for stress urinary incontinence. She develops acute urinary retention and fails two trials without catheter in 48 hours. What would you do next? (It can be assumed that she would either have an indwelling urethral catheter in situ or be using intermittent self-catheterisation in the meantime.)**
Loosen the tape at 3 days Loosen the tape at 7–14 days Divide the tape at 3 days Divide the tape at 14 days Divide the tape at 4 weeks Divide the tape at 6 weeks Do nothing Other: _____________________________________________________________

**Table 2:** Number of responses to each scenario

	1 response	2 responses	3 responses	4 responses	5 responses
Scenario 1	19	4	2	1	1
Scenario 2	19	4	2	1	1
Scenario 3	24	3	0	0	0
Scenario 4	23	4	0	0	0

## Results

**Figure 1:**
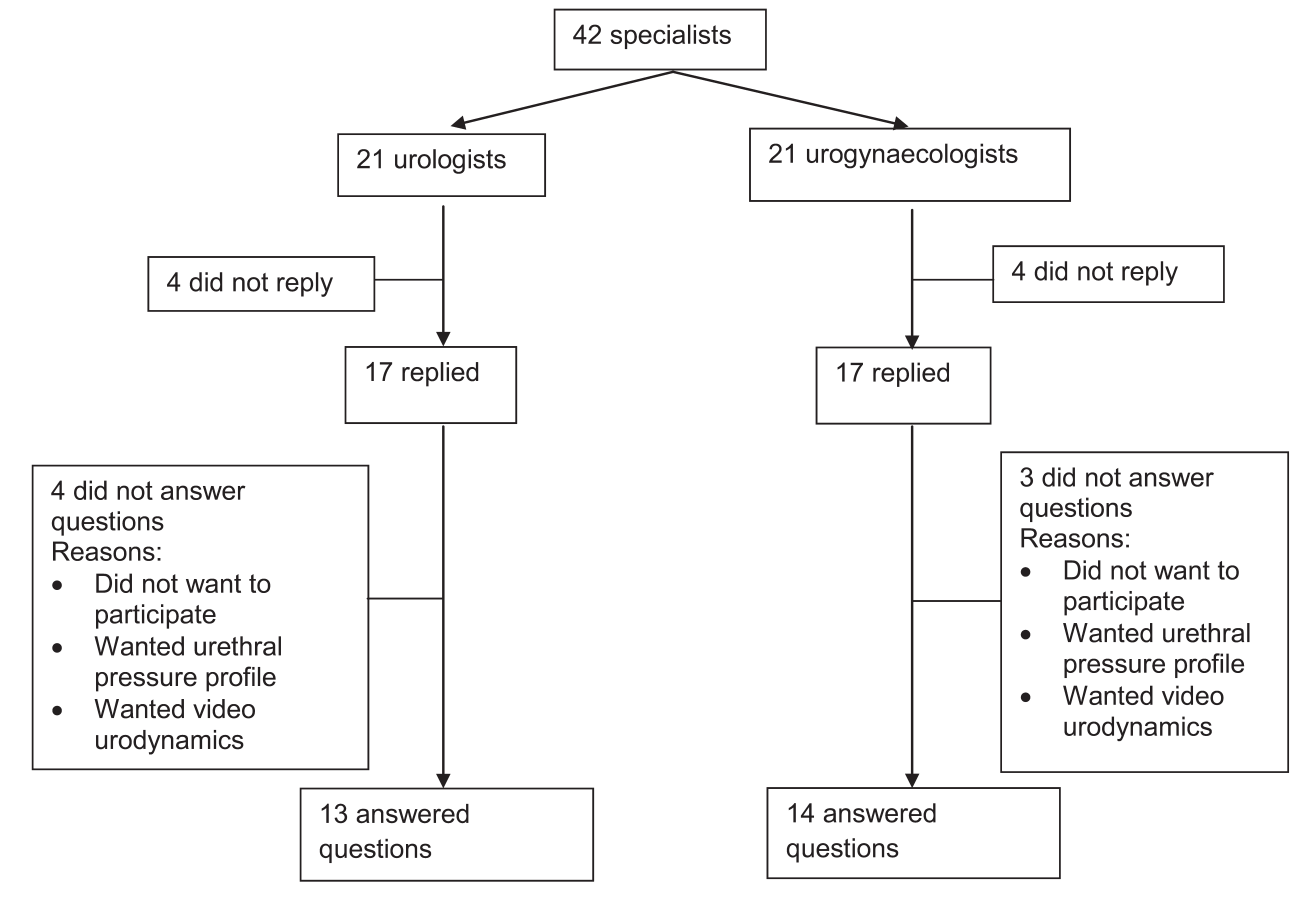
Flowchart of number of replies

Overall, 17 urologists (81%) and 17 urogynaecologists (81%) responded to the two emails. However, only 64% responded with answers to the questions (Fig 1). Those who responded to the emails but did not answer the questions replied saying they did not wish to participate in the survey or wanted further clinical information about the scenarios before being able to make a decision.

**Figure 2:**
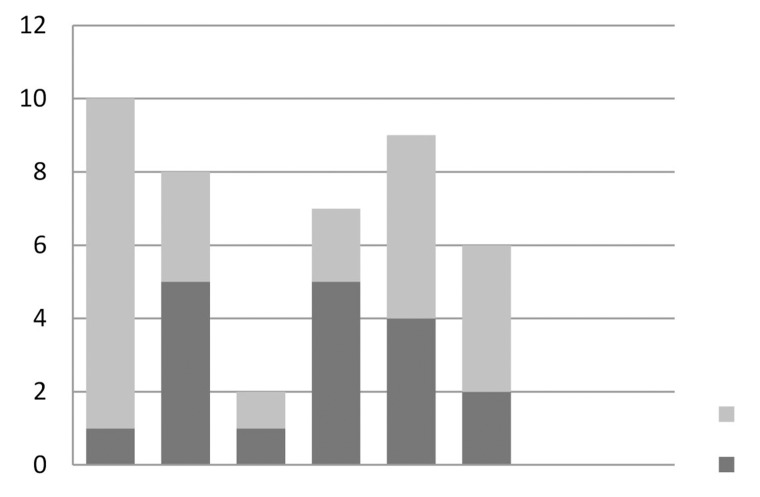
Replies to scenario 1

In scenario 1 the most common procedure performed in patients who continue to have SUI after a TVT was an insertion of another TVT (24%), with urogynaecologists being the majority choosing this option. The next most chosen options were colposuspension (21%) and TOT (19%), both of which were almost equally chosen between urologists and urogynaecologists (Fig 2).

**Figure 3:**
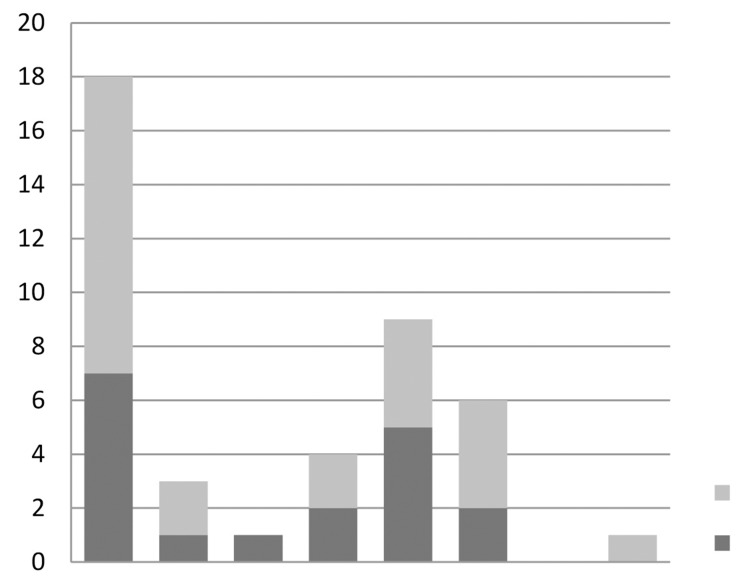
Replies to scenario 2

For scenario 2, in those who had a TOT as their first procedure, the majority opted for a TVT (43%) as their second procedure. The next most common option was colposuspension (21%) (Fig 3).

**Figure 4:**
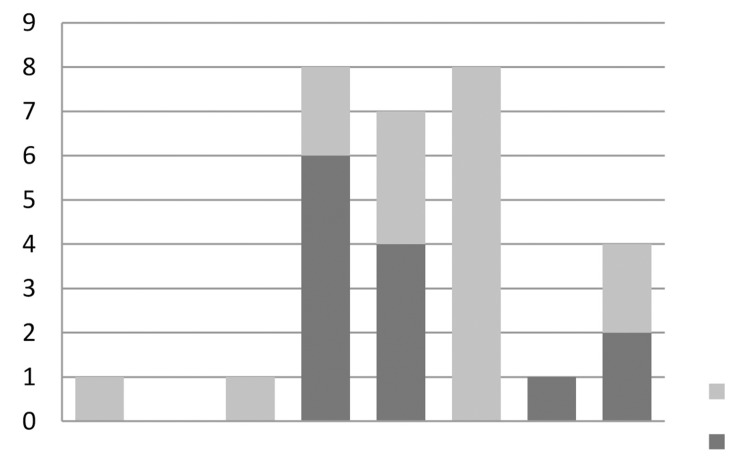
Replies to scenario 3

If a repeat TVT fails (scenario 3), the most common options for treatment were rectus sheath fascial sling (27%), periurethral injections (27%) and colposuspension (23%) (Fig 4). Interestingly, none of the urologists opted for a periurethral injection of bulking agent, with the majority choosing a rectus sheath fascial sling or colposuspension. In those who chose ‘other’, an artificial urinary sphincter was the choice option for 50% of respondents.

**Figure 5:**
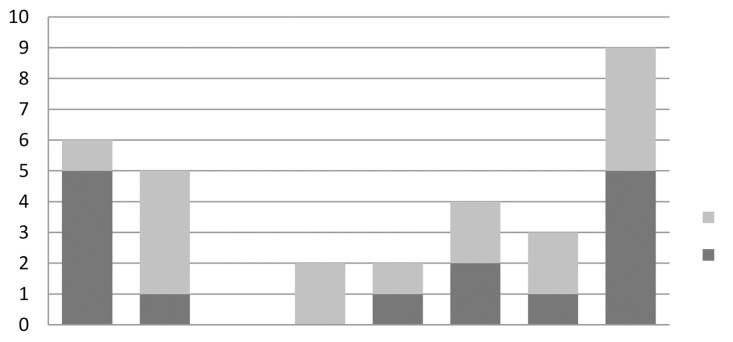
Replies to scenario 4

For those patients who had urinary retention (scenario 4), 35% of the respondents loosened the tape and 26% divided the tape, at various time scales (Fig 5). The ‘other’ responses included commencing clean intermittent self-catheterisation (CISC) for three, four or six months and then dividing the tape or just continuing CISC with no further management options given.

## Discussion

Midurethral sling procedures have become the most common surgery performed for SUI in women in England.^4^ However, this procedure is not without its complications. It is therefore important that the surgeons performing these procedures are able to manage poor post-operative outcomes appropriately as they impact considerably on a patient’s quality of life. In the absence of level 1 evidence from randomised controlled trials, dealing with these complications poses a dilemma to the surgeon.

Operations that have been reported in the literature for the management of recurrent SUI are based on retrospective cohort studies and include non-adjustable^6,7^ and adjustable^8,9^ synthetic slings, bulking agents,^10^ colposuspension,^11,12^ fascial slings,^13,14^ adjustable continence balloons^15^ and the artificial urinary sphincter.^16^ The choice of one over the other is difficult as it depends on several factors including clinician experience and patient preference.

Some experts would prefer to use advanced urodynamic testing before making a decision, including video urodynamics and urethral pressure profilometry to assess the shape/position of the bladder neck and urethra as well as to diagnose intrinsic sphincter deficiency or urethral hypermobility. Others would perform ultrasonography of the urethra^17^ to assess the position of the tape and to look at its dynamics. However, the advantage these tests offer over standard filling cystometry and pressure/flow studies has not been established in terms of effect on management and outcome of surgical intervention.

A repeat retropubic synthetic TVT seems to be the most favoured choice among experts for those patients who have already had a failed retropubic TVT. This finding was similar to that of a 2011 publication looking at the British Society of Urogynaecology database where 54% of patients with recurrent SUI had a repeat retropubic midurethral sling.^18^ Nevertheless, it must be emphasised that the cure rates after a second procedure tend to decline and the risk of *de novo* overactive bladder syndrome rises.^19^ A repeat retropubic TVT seems to offer better results than using a TOT following a failed primary TVT.

After failure of a second procedure for recurrent SUI, the majority of experts favoured either fascial slings, colposuspension or bulking agents. However, some said that a synthetic sling or an artificial urinary sphincter might be used.^20^ There are no data in the literature looking at this situation and a careful holistic review of the patient is certainly imperative before considering any further procedures. In such a case, a second opinion from a colleague may be invaluable as well as a discussion in a continence multidisciplinary meeting.

Management options for post-operative urinary retention include repeated voiding trials with urethral catheterisation in between trials of void, initiation of CISC, incision of the sling^21–23^ or pulling down the tape.^24,25^ Since many cases of post-operative voiding dysfunction will resolve spontaneously,^26^ the ideal timing for surgical intervention has not been defined. The clinician is therefore in a dilemma. On the one hand, early intervention may result in high rates of recurrent SUI in patients in whom the voiding dysfunction may have resolved spontaneously given enough time. Conversely, subjecting the patient to ongoing obstructive urinary tract symptoms, urinary tract infections or prolonged use of CISC is not ideal.

Some authors have recommended conservative therapy for post-operative voiding dysfunction for up to three months prior to attempting surgical intervention.^27^ However, a prolonged time to intervention for bladder outflow obstruction may be associated with long-term, potentially irreversible bladder dysfunction despite eventual successful relief of outflow obstruction.

Pulling down the tape within the first two weeks of surgery, dividing the tape between two and six weeks after the procedure or treating with CISC and dividing the tape after three to six months if the patient fails to void are all feasible options. Treatment will depend again on patient preference and careful counselling. The surgical conundrum is between relieving outflow obstruction or causing incontinence either if the tape is pulled down early or after division of the tape at a later date.

The introduction of adjustable tapes on the market may help in the reduction of recurrent incontinence and urinary retention. However, they will require full evaluation regarding success rates, complications and cost before they should be recommended.

## Conclusions

This is the first study that has attempted to gather expert opinion for the management of two common complications following surgery for SUI. It appears that there is no consensus among experts as to the best method of treating theses complications and it is therefore difficult to make any recommendations.

Nevertheless, the survey does show the urgent need for multicentre, randomised trials regarding the different management options of SUI and the complications of treating it. Failing this, the establishment of central databases that might direct clinicians towards best practice needs to be considered.
